# SNIP/p140Cap mRNA expression is an unfavourable prognostic factor in breast cancer and is not expressed in normal breast tissue

**DOI:** 10.1038/sj.bjc.6604365

**Published:** 2008-05-13

**Authors:** S Kennedy, M Clynes, P Doolan, J P Mehta, S Rani, J Crown, L O'Driscoll

**Affiliations:** 1 St Vincent's University Hospital, Dublin 4, Ireland; 2 National Institute for Cellular Biotechnology, Dublin City University, Dublin 9, Ireland

**Keywords:** breast cancer, SNIP, disease/relapse-free survival, overall survival, quantitative reverse-transcriptase polymerase chain reaction (qPCR)

## Abstract

The prevalence and clinical relevance of SNIP/p140Cap has not been extensively investigated. Here SNIP/p140Cap mRNA expression was studied in 103 breast tumour biopsies, where it was detected in ∼37% of tumour specimens, but not in any normal breast specimens. Expression correlated significantly with unfavourable overall survival. This suggests that SNIP/p140Cap may be a useful diagnostic and prognostic marker for breast cancer and its expression in breast cancer, but not in normal breast tissue, suggests that it may have potential as a therapeutic target.

Synaptosome-associated protein of 25 kDa (SNAP-25) was initially described as a neuronal membrane protein essential for neurite outgrowth and synaptic vesicle exocytosis (Chin *et al*, 2000). SNAP25-interacting protein (ie, SNIP), originally described as selectively expressed in the brain and co-distributed with SNAP-25, is tightly associated with the brain cytoskeleton and may serve as a linker protein connecting SNAP-25 to the submembranous cytoskeleton, where it is involved in regulating neurosecretion (Chin *et al*, 2000). Although NCBI described the human form of this transcript as homo sapiens SNAP-interacting protein (SNIP) mRNA (NM_025248), in general the rat form is described as SNIP (NP_062251), the mouse form as p140 (NP_061361), and sequence homology data suggest the human form to be the recently identified p130cas-associated protein, p140Cap (Di Stefano *et al*, 2004).

p130Cas, encoded by the *BCAR1* gene (van der Flier *et al*, 2001), is a signalling molecule involved in the linkage of actin cytoskeleton to the extracellular matrix during cell migration, cell invasion and transformation (Cabodi *et al*, 2004). Upon integrin engagement, p130Cas is tyrosine-phosphorylated and its presence has been shown to be required for integrin-dependent activation of epidermal growth factor receptor. It has been established that patients with primary breast tumours expressing a high level of p130Cas/BCAR1 protein, experience more rapid disease recurrence and are at a higher risk for intrinsic resistance to tamoxifen therapy (van der Flier *et al*, 2000; Cabodi *et al*, 2004). Transgenic mice overexpressing p130Cas in the mammary gland have been found to have extensive mammary epithelial hyperplasia, associated with activation of Src kinase, extracellular signal-regulated kinase 1/2, mitogen-activated protein kinase and Akt pathways, resulting in increased rates of proliferation and decreased apoptosis (Cabodi *et al*, 2006). Further evidence for the involvement of p130Cas in breast cancer include studies downregulating p130Cas expression with siRNA in her2-expressing cells that results in apoptosis, indicating p130Cas to be involved in cell survival. Immunohistochemical analysis of 150 human breast tumours indicated p130Cas overexpression in a high percentage of cases, independent of tumour histology type or grade (Cabodi *et al*, 2006).

Analysis of p140Cap, the p130CAS-associated protein (homo sapiens SNIP), has been limited to date. Immunoblotting studies indicated p140Cap expression in a range of cell lines, including human ECV304, T47 and HeLa, as well as rat FRT and murine N1E115 cells. Expression of p140Cap in NIH3T3 and ECV304 cells is reported to inhibit early phases of cell spreading on fibronectin. p140Cap involvement in integrin- and growth factor-mediated signalling, modulating the ability of cells to spread on matrix proteins, is proposed to be associated with p130Cas and actin stress fibres (Di Stefano *et al*, 2004). Western blot studies of adult mouse primary tissues indicate p140Cap expression in brain, testis and epithelial-rich tissues including lung, kidney and mammary glands (Di Stefano *et al*, 2004).

Here we report the first study of SNIP/p140Cap (SNIP) mRNA expression in a large bank of human specimens of any kind. We describe expression of this transcript in a retrospective series of 103 primary breast carcinomas and 19 normal breast tissue specimens, and we report how its presence is associated with outcome for breast cancer patients.

## MATERIALS AND METHODS

### Patients

Tissue specimens from 103 cases of primary breast cancers procured (snap-frozen within 30 min of procurement) during 1993–1997 at St Vincent's University Hospital, Dublin, were included in this study. A number of clinical and pathologic parameters were abstracted from patients’ charts including details on age, postoperative treatment and follow-up, tumour stage, and hormonal analysis. Tumours were typed, graded and staged as previously described. Nineteen noncancerous breast biopsies were also included in these studies to represent normal breast tissue.

### RNA extraction

Total RNA was isolated from all specimens using TriReagent (Sigma, Poole, England). RNA quantity and quality were assessed using a Nanodrop (ND-1000; Labtech International, East Sussex, UK) and an Agilent bioanalyser (Agilent 2100; Agilent Technologies, Santa Clara, CA, USA), respectively.

### cDNA formation on mRNA template

Following priming with oligo(dT) at 65°C for 5 min, followed by 1-min incubation on ice, cDNA was synthesised from 100 ng total RNA, using Superscript III RNase H-RNase OUT Ribonuclease (Invitrogen Biosciences Ltd., Dun Laoighare, Dublin) and a cocktail of dNTPs, by incubating at 50°C for 1 h, followed by 70°C for 15 min, in a 40 *μ*l reaction volume.

### Quantitative reverse-transcriptase PCR

The cDNA (diluted 1 : 10) was amplified in 25 *μ*l reactions by quantitative reverse-transcriptase PCR (qPCR), using an ABI 7500 Real-Time PCR System (Applied Biosystems, Foster City, CA, USA). Following evaluation of 11 potential endogenous controls in a random selection of 14 breast specimens (seven breast tumours and seven normal breast specimens), this study involved evaluation of SNIP/p140Cap mRNA in all 103 breast carcinomas and 19 normal breast specimens, in triplicate. Primers and TaqMan probes were designed using Primer Express Software 2.0, based on criteria applied by us in previous studies (Doolan *et al*, 2008). Primer and probe sequences for SNIP/p140Cap amplification were forward primer, 5′-AGACGCATCGTGCAACCTATG-3′; reverse primer, 5′-TGGCTGCGTCTCGGTATAGC-3′; probe, TCCAACCGCACAGGGCAGGGT. The profile of all reactions was 50°C for 2 min, 95°C for 10 min, 40 cycles of 95°C and 60°C for 1 min. Analysis including all components except cDNA (replaced with H_2_O) showed no contamination of reaction components. In addition, controls including RNA, but lacking reverse transcriptase enzyme and oligo(dT), respectively, verified no DNA/pseudogene contamination of starting material. SNIP/p140Cap threshold cycle (*C*_T_) results were subsequently normalised to two suitable endogenous controls – *β*-actin and glyceraldehyde 3-phosphate dehydrogenase (GAPDH) – and calibrated against a pooled cDNA from breast specimens, using the comparative *C*_T_ method, 2^−ΔΔ*C*T^ (Livak and Schmittgen, 2001).

### Statistical analysis

Statistical (univariate and multivariate) analyses of the results were performed using SPSS 12.1. The data were censored at 5 years for multivariate analysis. A value of *P*<0.05 was considered statistically significant.

## RESULTS

### Patient characteristics

The 103 consenting patients whose tumour biopsies (procured prior to any treatment with tamoxifen or chemotherapeutic agents) were included in this study were aged between 31 and 90 years at the time of diagnosis (mean age=58 years). Twenty-six women were <50 years and 77 were ⩾50 years of age at diagnosis. Tumours ranged from 0.6 to 8.0 cm (mean=2.8 cm). Eighteen tumours were T1; 82 tumours were T2 and three tumours were T3. Eighty-one tumours were invasive ductal carcinoma, 17 were invasive lobular and five were tumours of special type (two tubular and three mucinous). Eleven tumours were grade 1; 31 were grade 2; and 53 were grade 3. Sixty-six tumours were ER^+^ and 44 were ER^−^. Oestrogen receptor status was not available for three patients. Forty-five tumours had no axillary metastases, whereas 58 had metastasised to axillary lymph nodes.

Sixty-nine women were treated with postoperative tamoxifen; 25 did not receive tamoxifen. Forty-nine patients were treated with adjuvant systemic chemotherapy (CMF+/−adriamycin); 45 patients did not receive chemotherapy. Details regarding tamoxifen and systemic chemotherapy were not available for nine patients. Maximal follow-up was 3026 days with a mean follow-up of 1887 days.

### Quantitative reverse-transcriptase PCR

Analysis for 11 potential endogenous controls in a random selection of 14 (seven tumour; seven normal) specimens indicated 18S, *β*_2_-microglobin, *β*-actin and GAPDH mRNAs to be expressed at similar levels in all specimens analysed. *β*-actin and GAPDH were subsequently selected for amplification in all specimens, in parallel with SNIP/p140Cap, and the mean of their *C*_T_ values was used for normalisation.

### Detection of SNIP/p140Cap mRNA

SNIP/p140Cap mRNA was detected in 36.9% (38/103) of the breast tumour specimens analysed and was found not to be expressed in any of the normal breast specimens.

### Prognostic relevance of SNIP/p140Cap

The prognostic relevance of SNIP/p140Cap expression at diagnosis was evaluated in relation to relapse/disease-free survival (RFS) and overall survival (OS).

In relation to RFS, Cox univariate analysis of RFS (Table 1) indicated that expression of SNIP/p140Cap transcript did not associate with RFS, whereas tumour size (*P*=0.009) and grade (*P*=<0.0005), treatment with adjuvant chemotherapy (*P*=0.002), lymph-node status (*P*<0.0005) and ER status (*P*=0.004) showed significant correlation with RFS. These results were supported by Kaplan–Meier analysis (Table 1), indicating ER positivity to be a favourable factor (*P*=0.0032), with increased tumour size (*P*=0.007), advanced grade (*P*=0.0013), treatment with adjuvant chemotherapy (*P*=0.0012) and spread to lymph nodes (*P*<0.00005) to correlate with bad prognosis.

**Figure 1:**
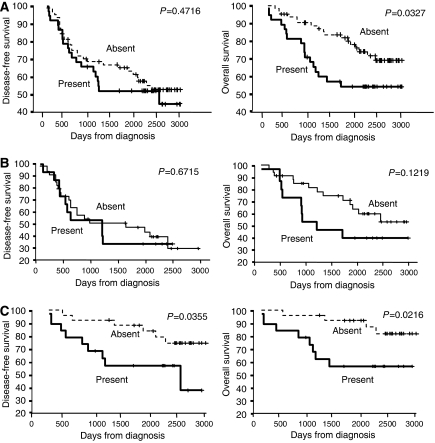
(**A**) Kaplan–Meier survival curves for SNIP mRNA presence or absence and its association with disease/relapse-free survival (RFS) and overall survival (OS), respectively. A significant unfavourable association was found between expression of SNIP mRNA and OS. (**B**) SNAP25-interacting protein expression did not show a significant association with RFS and OS, respectively, for patients who received adjuvant chemotherapy, but (**C**) showed significant association with RFS and OS, respectively, for patients who did not receive adjuvant chemotherapy.

Chi-squared analysis indicated a significant association between SNIp/P140Cap mRNA expression and tumour size (*P*=0.02) and tamoxifen therapy (*P*=0.037) (see Table 2). No other significant associations were found between expression of SNIP/p140Cap and age at diagnosis, tumour grade, tumour type, treatment with adjuvant chemotherapy, lymph-node status or ER status. By multivariate analysis (Table 3), the most important prognostic factors for RFS were found to be spread of cancer to lymph nodes (*P*<0.0005), ER status (*P*=0.001), tumour size (*P*=0.011) and tumour grade (*P*=0.045). Kaplan–Meier analysis indicated no correlation between RFS and expression of SNIP/p140Cap mRNA (Figure 1A).

Univariate Cox analysis (Table 1) indicated that the factors investigated in this study that were significant prognostic indicators of OS were SNIP/p140Cap mRNA expression (*P*=0.037), tumour size (*P*=0.048), tumour grade (*P*<0.0005), tumour type (*P*=0.031) and lymph-node status (*P*=0.001). Expression of ER was strongly associated with OS; however, statistical significance was not reached (*P*=0.056). These results were supported by Kaplan–Meier analysis, indicating SNIP/p140Cap expression (*P*=0.0327), increased tumour size (0.0437), tumour type (invasive ductal) (*P*=0.0453), advanced stage of tumours (*P*=0.0003), adjuvant chemotherapy (*P*=0.0457) and spread to lymph nodes (*P*=0.0006) to be significantly correlated with poor prognosis. No significant association was found between OS and age at diagnosis or adjuvant tamoxifen treatment. Multivariate analysis (Table 3) indicated lymph-node status (*P*=0.003), tumour grade (*P*=0.017) and SNIP/p140Cap mRNA expression (*P*=0.005) to be independent prognostic factors for OS. The unfavourable association of SNIP/p140Cap mRNA expression (*P*=0.0327) with outcome for patients, indicated by Kaplan–Meier analysis, is shown in Figure 1A.

### Predictive relevance of SNIP/p140Cap expression

To establish if expression of SNIP/p140Cap mRNA is predictive of response to adjuvant chemotherapy, Kaplan–Meier analysis was performed on those cases only where chemotherapy was administered. As indicated in Figure 1B, the results of this analysis suggest SNIP/p140Cap expression not to be predictive of outcome in terms of either RFS or OS. Interestingly, analysis of cases where chemotherapy was not administered indicated SNIP/p140Cap mRNA expression to be associated with poor outcome for patients in terms of both RFS (*P*=0.0355) and OS (*P*=0.0216) (Figure 1C).

## DISCUSSION

This study represents the first reported analysis of SNIP/p140Cap expression and relevance to patients’ outcome in cancer. SNIP/p140Cap mRNA was detected in approximately 37% of the breast tumours analysed, and its expression was significantly associated with increased tumour size. Although an association was not found between expression of SNIP/p140Cap mRNA and RFS, a significant association was evident between expression of this transcript and disease outcome, in terms of OS, with the presence of SNIP/p140Cap mRNA associated with shorter survival from diagnosis. Indeed, multivariate analysis indicates that, similar to advanced tumour grades and spread of cancer to lymph nodes, SNIP/p140Cap mRNA expression is an independent unfavourable prognostic factor for OS. Whether or not the *in vivo* effects of SNIP/p140Cap are due to its interactions with p130Cas is yet to be determined. However, unfavourable associations between p130Cas expression and breast cancer recurrence and resistance to tamoxifen (van der Flier *et al*, 2000, 2001) and our identification (as described here) of SNIP/p140Cap in breast tumours – but not in normal breast tissue – and its association with larger tumours and shorter survival times from diagnosis, suggest that its effects may, at least in part, be due to an involvement with p130Cas.

The results from this study were further analysed to investigate a potential predictive relevance for SNIP/p140Cap. Although a significant association was not found between expression of this transcript and outcome for patients who received adjuvant chemotherapy, we identified the presence of SNIP/p140Cap to be associated with unfavourable outcome, in terms of both RFS and OS, for patients who did not receive chemotherapy.

These novel findings suggest that SNIP/p140Cap analysis by qPCR may have potential as both a diagnostic and a prognostic biomarker study for breast cancer. Its expression in breast tumours, but not in normal tissue, suggests that it may have potential as a therapeutic target.

## Figures and Tables

**Table 1:** Univariate Cox analysis supported by Kaplan–Meier analysis

	**Overall survival (OS)**		**Relapse-free survival (RFS)**	
**Characteristics**	***P* a**	***P* b**	***P* a**	***P* b**
Age (<50 *vs* ⩾50 years.)	0.964	0.9636	0.263	0.2599
Tumour size (<2.8 *vs* ⩾2.8 cm)	0.048^*^	0.0437^*^	0.009^*^	0.007^*^
Lymph-node metastasis (negative *vs* positive)	0.001^*^	0.0006^*^	<0.0005^*^	<0.00005^*^
Histology grade (I and II *vs* III)	<0.0005^*^	0.0003^*^	<0.0005^*^	0.0013^*^
Histology type (IDC *vs* ILC *vs* special)	0.031^*^	0.0453^*^	0.206	0.0624
ER status (negative *vs* positive)	0.056	0.0514	0.004^*^	0.0032^*^
Chemotherapy (yes *vs* no)	0.050	0.0457^*^	0.002^*^	0.0012^*^
Tamoxifen (yes *vs* no)	0.449	0.4475	0.182	0.1784
SNIP (absent *vs* present)	0.037^*^	0.0327^*^	0.473	0.4716

IDC=invasive ductal carcinoma; ILC=invasive lobular carcinoma; SNIP=SNAP25-interacting protein.

^*^Significant parameter.

Mean size (2.8 cm) was used as cutoff; grades I and II grouped together *vs* grade III. Kaplan–Meier analysis supports the univariate Cox regression studies, indicating ER positivity to be associated with favourable prognosis whereas expression of SNIP mRNA, increased tumour size, tumour type, advanced grade, spread to nodes and adjuvant chemotherapy treatment are associated with poor outcome for patients.

a Cox regression *P*-value.

b Kaplan–Meier *P*-value.

**Table 2:** Correlation between clinicopathological factors and expression of SNIP mRNA in breast carcinoma

**Characteristics**	**No. of cases**	**SNIP (absent *vs* present) (%)**	***P***
*Age (years)*			
<50	8/26	30.8	0.454
>50	30/77	39.0	
			
*Tumour size*			
<2.8 cm	15/56	26.8	0.020^*^
>2.8 cm	23/47	48.9	
			
*Lymph-node metastasis*			
Negative	17/45	37.8	0.870
Positive	21/58	36.2	
			
*Histology grade*			
I	6/11	54.5	0.347
II	12/39	30.8	
III	20/53	37.7	
			
*Histology type*			
IDC	32/81	39.5	0.458
ILC	4/17	23.5	
Special	2/5	40.0	
			
*ER status*			
Negative	13/34	38.2	0.738
Positive	23/66	34.8	
			
*Chemotherapy*			
No	19/45	42.2	0.242
Yes	15/49	30.6	
			
*Tamoxifen*			
No	5/25	20.0	0.037^*^
Yes	30/69	43.5	

IDC=invasive ductal carcinoma; ILC=invasive lobular carcinoma; SNIP=SNAP25-interacting protein.

^*^Significant parameter.

*P*-values from *χ*^2^ analyses.

**Table 3:** Multivariate Cox regression backward stepwise (likelihood ratio)

**Characteristics**	**Overall survival (OS)**	**Relapse-free survival (RFS)**
	*P*	*P*
Lymph node (spread *vs* no spread)	0.003	<0.0005
ER (absence *vs* presence)	NS	0.001
Histology grade (I and II *vs* III)	0.017	0.045
Tumour size (<2.8 *vs* ⩾2.8 cm)	NS	0.011
SNIP mRNA (absence *vs* presence)	0.005	NS

NS=not significant; SNIP=SNAP25-interacting protein.

Parameters in the multivariate analysis included age, tumour size, tumour grade, lymph-node status, ER status, as well as SNIP mRNA expression. Mean size (2.8 cm) was used as cutoff; grade I and II grouped together *vs* grade III. This table summarises significant factors.
